# Retinoids cause apoptosis in pancreatic cancer cells via activation of RAR-γ and altered expression of Bcl-2/Bax

**DOI:** 10.1038/sj.bjc.6600496

**Published:** 2002-08-27

**Authors:** F Pettersson, A G Dalgleish, R P Bissonnette, K W Colston

**Affiliations:** Department of Oncology, Gastroenterology, Endocrinology and Metabolism, St George's Hospital Medical School, London SW17 ORE, UK; Ligand Pharmaceuticals Inc., San Diego, California, CA 92121, USA

**Keywords:** pancreatic cancer, retinoids, apoptosis, Bcl-2

## Abstract

All-*trans*-retinoic acid and 9-*cis*-retinoic acid have been reported to have inhibitory effects on pancreatic adenocarcinoma cells and we have shown that this is partly due to induction of apoptosis. In this study, the mechanisms whereby 9-*cis*-retinoic acid induces apoptosis in these cells were investigated. An involvement of the Bcl-2 family of proteins was shown, such that 9-*cis*-retinoic acid causes a decrease in the Bcl-2/Bax ratio. Overexpression of Bcl-2 also resulted in inhibition of apoptosis induced by 9-*cis*-retinoic acid. Furthermore, two broad-range caspase inhibitors blocked DNA fragmentation induced by 9-*cis*-retinoic acid, but had no effect on viability defined by mitochondrial activity. Using synthetic retinoids, which bind selectively to specific retinoic acid receptor subtypes, we further established that activation of retinoic acid receptor-γ is essential for induction of apoptosis. Only pan-retinoic acid receptor and retinoic acid receptor-γ selective agonists reduced viability and a cell line expressing very low levels of retinoic acid receptor-γ is resistant to the effects of 9-*cis*-retinoic acid. A retinoic acid receptor-β/γ selective antagonist also suppressed the cytotoxic effects of 9-*cis*-retinoic acid in a dose-dependent manner. This study provides important insight into the mechanisms involved in suppression of pancreatic tumour cell growth by retinoids. Our results encourage further work evaluating the clinical use of receptor subtype selective retinoids in pancreatic carcinoma.

*British Journal of Cancer* (2002) **87**, 555–561. doi:10.1038/sj.bjc.6600496 www.bjcancer.com

© 2002 Cancer Research UK

## 

Despite a lot of effort put into improving diagnosis, staging and treatment of pancreatic cancer during the past decades, it is still one of the leading causes of cancer-related deaths in the western world. Five-year survival is less than 5% and the median survival time for patients diagnosed with advanced disease is about 5 months (Rosewicz and Wiedenmann, 1997; Harris and Bruckner, 2001). Surgery is the only curative treatment for pancreatic cancer but a very small percentage of tumours are actually resectable, due mainly to the fact that most patients present late with locally advanced or metastatic disease. Even after surgery, 5-year survival rates reported from most institutions are only around 10% (Rios *et al*, 1999; Wagner *et al*, 1999) and as a consequence, adjuvant and neoadjuvant treatments are important issues. Current chemotherapeutic strategies are generally ineffective (van Riel *et al*, 1999).

Retinoids are natural or synthetic derivatives of vitamin A. The physiological form of vitamin A is retinol, which is metabolised in its target cells to retinal and then further oxidised to form all-*trans*-retinoic acid (ATRA) and its stereoisomer 9-*cis*-retinoic acid (9cRA) (Vieira *et al*, 1995). In the late sixties, vitamin A was shown to have anti-tumour activity (Saffiotti *et al*, 1967) and it has since been shown that retinoic acids and other retinoid-related compounds cause growth inhibition, accompanied by induction of differentiation and/or apoptosis, in various types of cancer cells (Bollag *et al*, 1994; de Luca *et al*, 1995; Lippman *et al*, 1995). Retinoids exert their effects by interacting with nuclear receptors functioning as ligand-dependent transcription factors that switch a variety of genes on and off (Evans, 1988). There are two families of receptors binding retinoic acids, the retinoic acid receptors (RARs) and the retinoid X receptors (RXRs). Each family has three subtypes (α, β and γ) and each of those a number of isoforms. The natural ligand for RARs is ATRA whereas 9cRA binds to both RARs and RXRs with high affinity (Heyman *et al*, 1992). The activity of RARs and RXRs is further regulated by the presence of numerous coregulatory molecules, which act at least in part through regulation of histone acetylation and modulation of chromatin structure (for review see Freedman, 1999; Glass and Rosenfeld, 2000).

Responsiveness to retinoids in pancreatic cancer cells has previously been reported by different groups (Rosewicz *et al*, 1995; Bold *et al*, 1996; Louvet *et al*, 1996). A phase II trial of 13-cis retinoic acid and interferon-α in patients with advanced pancreatic carcinoma has also been completed and promising results with one partial remission and 14 out of 22 patients with stable disease for a median duration of 5 months were reported (Brembeck *et al*, 1998). We have shown that ATRA and 9cRA induce apoptosis in a number of pancreatic adenocarcinoma cell lines and enhance the cytotoxic effects of chemotherapeutic agents in these cells (Pettersson *et al*, 2000, 2001). In the present study, we examined the role of two families of proteins that are important players in many apoptotic responses, the Bcl-2 proteins and caspases. Furthermore, the involvement of different RAR and RXR subtypes in this response was investigated and a critical role for RAR-gamma was identified.

## MATERIALS AND METHODS

### Cell lines

The human pancreatic adenocarcinoma cell line T3M-4 was obtained from Professor N Lemoine (ICRF, UK). All other cells were obtained from the American Tissue Culture Collection. Cells were maintained in RPMI-1640 culture medium with 10% foetal calf serum (FCS), 2 mM L-glutamine and antibiotics at 37°C in a humidified atmosphere containing 5% CO_2_. During all experiments, cells were grown in medium containing 2.5% FCS. Under these conditions, the level of endogenous retinoic acid in the medium is less than 10^−9^ M, which is negligible (Napoli, 1996). Cells were routinely tested for mycoplasma contamination.

### Reagents

ATRA and 9cRA were purchased from SIGMA (Poole, UK). Receptor subtype-selective analogues were provided by Ligand Pharmaceuticals Inc. (San Diego, CA, USA) The compounds were characterised for their receptor subtype selectivity as previously described (Allegretto *et al*, 1993) (see Table 1

**Table 1:**
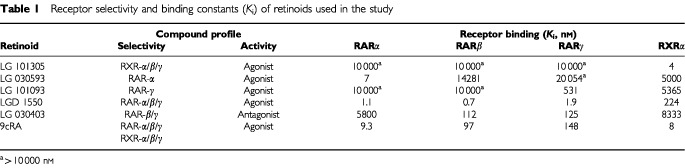
Receptor selectivity and binding constants (*K*_i_) of retinoids used in the study

). The pUSEamp(+) plasmid containing wild-type mouse Bcl-2 under the control of the cytomegalovirus (CMV) promoter was obtained from Upstate Biotechnology (Lake Placid, NY, USA). A control vector without insert was supplied by the same source. The broad range caspase inhibitors Z-VAD.fmk (Z-Val-Ala-Asp(OMe)-CH_2_F) and Boc-D.fmk (Boc-Asp(OMe)-CH_2_F) were purchased from Calbiochem (Nottingham, UK). The peptides were dissolved in DMSO and stock solutions kept at −20°C. Antibodies for RAR-α, β, γ, RXR-α and Bax were purchased from Santa Cruz Biotechnology, Inc. (Santa Cruz, CA, USA). A Bcl-2 specific antibody was obtained from Dako (High Wycombe, UK).

### Viability assay

Assessment of relative numbers of viable cells was done using an MTT tetrazolium assay (Mosmann, 1983). Cells were cultured in 96-well plates in 200 μl of medium containing 2.5% serum and the inhibitory compounds. At each time point, 20 μl 3-(4,5-dimethylthiazol-2-yl)-2,5-diphenyltetrazolium bromide (MTT) (5 mg ml^−1^, SIGMA) was added to each well and the plates were incubated at 37°C for 4 h. The medium was then removed and formazan crystals were dissolved in 100 μl of acidic isopropanol (0.05 M HCl). Optical density was measured at 550 nm.

### Apoptosis assays

Propidium iodide (PI) staining followed by flow cytometric analysis was used to detect cells with a sub-G1 DNA content. After treatment, cells were harvested by trypsinisation, washed twice with sample buffer (PBS+1 g l^−1^ glucose) and fixed in 70% ethanol at a density of 1×10^6^ cells/ml. After ⩾18 h cells were washed with sample buffer and resuspended in PI staining solution containing 50 μg ml^−1^ PI and 20 μg ml^−1^ RNase A. Fluorescence was measured on a Becton-Dickinson FACScan and DNA histograms were analysed using ModFitLT software. Apoptosis was also assessed using the Boeringer-Mannheim Cell Death Detection ELISA^PLUS^ kit, which detects the presence of histone-associated DNA fragments in the cell cytosol, according to the manual supplied by the manufacturer. Apoptotic index=OD_(treated cells)_/OD_(untreated cells)_. The two methods gave comparable results.

### Stable transfections

T3M-4 cells were transfected with the pUSEamp.Bcl-2 plasmid or a control plasmid without insert, using SuperFect™ transfection reagent (Qiagen Ltd., West Sussex, UK), according to the manufacturer's instructions. Selection of transfected clones was done using culture medium containing 0.6 mg ml^−1^ G418 sulphate (Geneticin®, Life Technologies, Paisley, UK). Expression of Bcl-2 was assessed by Western blotting in selected clones using an antibody supplied by Upstate Biotechnology (Lake Placid, NY, USA).

### Western blotting

Cells were harvested by scraping and whole cell lysates were prepared by washing the cells in ice cold PBS and resuspending them in lysis buffer (50 mM Trizma, pH 8.0, 150 mM NaCl, 0.1% Triton X-100, 0.01 mg ml^−1^ aprotinin, 0.05 mg ml^−1^ PMSF), followed by sonication on ice and ultracentrifugation. Equal amounts of protein were electophoretically separated in 10–12% SDS polyacrylamide gels and proteins were immobilised by transfer onto nitrocellulose membranes. Membranes were immunoprobed with the relevant antibodies followed by a secondary, peroxidase-labelled antibody. The proteins of interest were visualized using a luminescent visualization system (HRPL™, National Diagnostics, Hull, UK).

### Statistics

All experiments were performed at least twice and results shown are means of all determinations unless otherwise stated. Statistical comparisons were made using an unpaired, two-tailed *t*-test or ANOVA followed by the Fisher PLSD test (StatView 4.0 software package for Apple Macintosh). All comparisons are made relative to an untreated control, unless otherwise stated, and significance is indicated as *P<0.05, **P<0.01, ***P<0.005.

## RESULTS

### Loss of cell viability and induction of apoptosis

**Figure 1:**
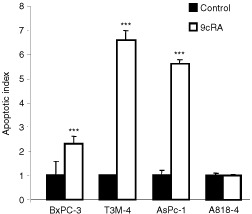
Assessment of apoptosis by cell death detection ELISA. The cells were treated with 500 nM 9cRA for 6 days and results are expressed as means±s.d. (*n*=4).

We have previously shown that treatment of pancreatic adenocarcinoma cells with ATRA or 9cRA for 6 days results in a dose-dependent reduction in the number of viable cells. We show here that 9cRA induced apoptosis in BxPc-3, T3M-4 and AsPc-1, but not A818-4 pancreatic adenocarcinoma cells (Figure 1

).

### Suppression of apoptosis by Bcl-2

**Figure 2:**
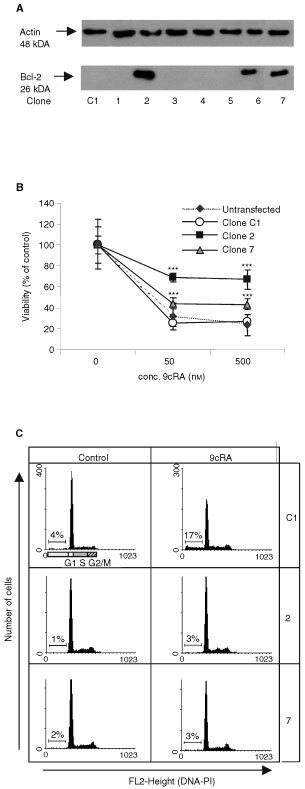
(**A**) Western analysis of Bcl-2 expression in transfected T3M-4 cells. Clone C1 is transfected with the control vector. (**B**) The inhibitory effect of 9cRA is significantly suppressed in cells overexpressing Bcl-2. Viability was assessed on day 6 of treatment, using an MTT assay. Results are expressed as means±s.d. (*n*=6). (**C**) Propidium iodide staining and cell cycle analysis confirm that apoptosis is inhibited. Representative histograms are shown and percentages are means of duplicate treatments. Cells were treated with 500 nM 9cRA for 6 days.

**Figure 3:**
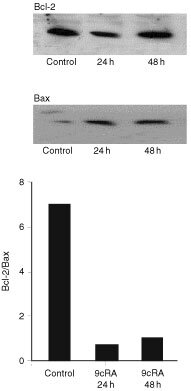
The Bcl-2/Bax ratio is decreased in parental T3M-4 cells after treatment with 500 nM 9cRA. Protein levels were assessed by semi-quantitative Western blotting and laser densitometry was used to quantify the signals. A representative blot is shown and the densitometric values are means of two determinations.

All cells in our study expressed moderate levels of Bcl-2 (not shown). To assess if enhanced expression of this anti-apoptotic protein would affect the ability of 9cRA to induce apoptosis, T3M-4 cells were transfected with the pUSEamp.Bcl-2 plasmid. This resulted in generation of three clones that stably over-expressed Bcl-2 (Figure 2A

). Two of these, clones 2 and 7, and the control transfected clone C1 were used in all subsequent experiments. We show that the apoptosis-inducing effect of 9cRA is suppressed by Bcl-2, as clone 2 and 7 were significantly less sensitive than clone C1 or parental, untransfected T3M-4 cells. Following treatment with 500 nM 9cRA for 6 days, loss of cell viability, as determined using MTT, was decreased and no apoptosis could be detected (Figure 2B,C). Furthermore, expression levels of Bcl-2 and Bax in parental T3M-4 cells were examined by semi-quantitative Western blotting in untreated cells and on day 2 and 4 of treatment with 500 nM 9cRA. A decrease in the Bcl-2/Bax ratio was found in treated cells, and this was due to decreased levels of Bcl-2 as well as increased levels of Bax (Figure 3

). 9cRA also caused a slight, non-significant decrease in the level of the anti-apoptotic Bcl-2 family member Mcl-1 (data not shown).

### Effects of caspase inhibition

**Figure 4:**
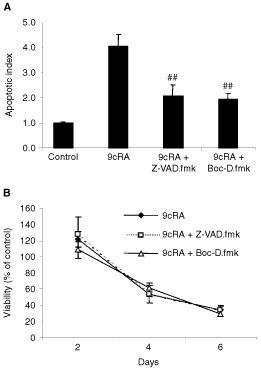
The broad-range caspase inhibitors Z-VAD.fmk and Boc-D.fmk specifically inhibited DNA fragmentation induced by 9cRA (**A**), but had no effect on mitochondrial activity (**B**). T3M-4 cells were treated with 500 nM 9cRA±50 μM Z-VAD.fmk or 25 μM Boc-D.fmk for 6 days and DNA fragmentation and viability were assessed using the Cell Death Detection ELISA (*n*=4) and an MTT assay (*n*=6), respectively. ^##^Indicates a significant difference compared to 9cRA alone (*P*<0.01).

Cells were preincubated with the broad-range caspase inhibitors Z-VAD.fmk or Boc-D.fmk for 3 h before addition of 9cRA. They were subsequently treated for up to 6 days and fresh medium, containing both compounds, was added every 48 h. Addition of either of the inhibitors resulted in a significant decrease in nuclear fragmentation, as determined by Cell Death Detection ELISA on day 6. Interestingly, mitochondrial activity was not affected, and no increase in cell viability was seen at any time point (Figure 4

).

### Receptor expression

**Figure 5:**
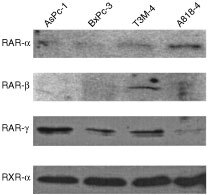
Semi-quantitative Western blots showing expression of RAR-α, -β and -γ and RXR-α in AsPc-1, BxPc-3, T3M-4 and A818-4 pancreatic adenocarcinoma cell lines. Equal amounts of total protein was loaded on the gel, based on the Bradford assay.

Expression of RAR-α, β and γ and RXR-α was assessed in AsPc-1, BxPc-3, T3M-4 and A818-4 cells, using semi-quantitative Western blotting. Weak expression of RAR-α was detected in the four cell lines, whereas very weak expression of RAR-β could be detected in T3M-4 only. RAR-γ was expressed in AsPc-1, BxPc-3 and T3M-4 cells, but only very weakly in 9cRA-resistant A818-4 cells. Approximately equal levels of RXR-alpha were seen in all four cell lines (Figure 5

). Using RT–PCR or Western blotting, we could detect no induction of receptor expression upon retinoid treatment (data not shown).

### Role of RAR and RXR subtypes in apoptosis

**Figure 6:**
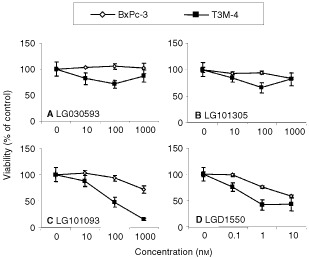
Reduced cell viability is seen in BxPc-3 and T3M-4 cells treated with the RAR-γ selective agonist LG101093 (**C**) and the pan-RAR agonist LGD1550 (**D**), but not with the RAR-α selective agonist LG030593 (**A**) or the pan-RXR selective agonist LG101305 (**B**). Per cent viable cells, compared to untreated control cells, was determined on day 6 using an MTT assay and results are expressed as means±s.d. (*n*=6).

**Figure 7:**
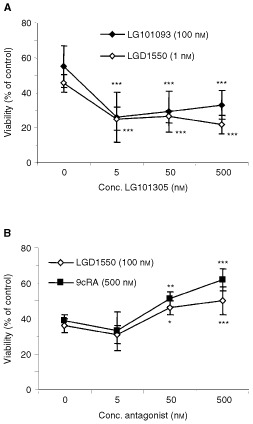
(**A**) Cotreatment with LG101093+LG101305 or LGD1550+LG101305 results in enhanced loss of cell viability. T3M-4 ells were treated with LG101093 or LGD1550 plus increasing concentrations of LG101305 for 6 days and results are expressed as means±s.d. (*n*=12). (**B**) The RAR-β/γ selective antagonist LG030403 suppresses the inhibitory effect of LGD1550 and 9cRA in a dose-dependent manner. T3M-4 cells were treated with LGD1550 or 9cRA plus increasing concentrations of LG030403 for 6 days. Cell viability was determined using MTT and results are expressed as means±s.d. (*n*=6). Per cent of control means relative to untreated cells.

To assess the role of receptor subtypes in retinoid-induced apoptosis in our cells, we treated T3M-4 and BxPc-3 with subtype selective retinoids for up to 6 days. We show that only two of the compounds, the RAR-γ selective LG101093 and the pan-RAR agonist LGD1550, caused reduced cell viability. As previously reported (Shalinsky *et al*, 1997), LGD1550 was significantly more potent than any of the other compounds, with an IC_50_ value which is around 500-fold lower than the IC_50_ value of LG101093. As single agents, the pan-RXR selective LG101305 and the RAR-α selective LG030593 had little or no effects (Figure 6

). However, addition of LG101305 to LG101093 or LGD1550 resulted in significantly increased inhibition of cell viability (Figure 7A

), although this was only observed at relatively low levels of LG101093 and LGD1550. Co-treatment with LG030593 plus LG101093 did not confer any additional inhibition, compared to LG101093 alone (not shown). Importantly, addition of an RAR-beta/gamma selective antagonist, LG030403, resulted in significant suppression of the effects of LGD1550 as well as 9cRA (Figure 7B)

**Figure 8:**
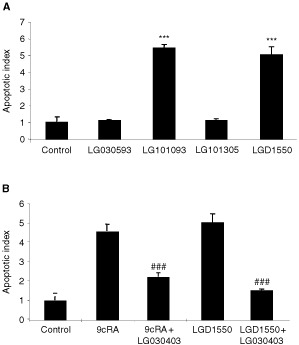
LG101093 and LGD1550 induce apoptosis in T3M-4 cells as determined by the Cell Death Detection ELISA. Cells were treated for 6 days with 10 nM of LGD1550 and 500 nM of each of the other compounds. (**B**) The RAR-β/γ selective antagonist LG030403 inhibits apoptosis induced by 9cRA and LGD1550. Cells were treated for 6 days with 500 nM 9cRA or 10 nM LGD1550, with or without 500 nM LG030403. Results are means±s.d. (*n*=4). ^###^ Indicates a significant difference compared to agonist alone (*P*<0.005).

Using the Cell Death Detection ELISA, it was confirmed that LG101093 and LGD1550 induce apoptosis in T3M-4 cells, causing significant nuclear fragmentation. Neither of the other compounds had this effect. Again, LGD1550 was significantly more potent, causing the same level of apoptosis as LG101093 and 9cRA at a 50-fold lower concentration (Figure 8A,B

). Addition of the RAR-β/γ antagonist, LG030403, resulted in significant suppression of the effects of LGD1550 as well as 9cRA (Figure 8B).

## DISCUSSION

ATRA and 9cRA have previously been reported to have anti-proliferative effects and to modulate differentiation in pancreatic cancer cells (Rosewicz *et al*, 1995; Bold *et al*, 1996; Louvet *et al*, 1996). We have shown that they can also act as potent apoptosis-inducers (Pettersson *et al*, 2000), and in this study we have further elucidated the mechanisms behind this finding.

The Bcl-2 family of pro- and anti-apoptotic proteins plays an important role in apoptosis induced by a large variety of stimuli (Gross *et al*, 1999). We show here that overexpression of Bcl-2 in T3M-4 cells efficiently inhibits induction of apoptosis by 9cRA. Treatment of parental T3M-4 cells with 9cRA also causes a decrease in the Bcl-2/Bax ratio, which may facilitate cell death. It may be hypothesised that this is important for the ability of retinoids to enhance the sensitivity of pancreatic cancer cells to other cytotoxic drugs (Pettersson *et al*, 2001). Until now, the relationship between apoptosis induced by retinoic acid and expression of Bcl-2 has been explored mainly in leukaemic cells. In agreement with the results presented here, down-regulation of Bcl-2 expression by retinoids has been observed in acute promyelocytic and myeloid leukaemic cells, and stable overexpression of Bcl-2 has been shown to confer resistance to apoptosis (Nagy *et al*, 1996; Bocchia *et al*, 1997; Bruel *et al*, 1997).

In recent years, several reports have also suggested that the sub-cellular localisation of Bcl-2 may determine its function. For example, stable overexpression of Bcl-2 targeted to the endoplasmatic reticulum (ER) is protective against apoptosis induced by radiation as well as serum starvation, but does not inhibit apoptosis induced by etoposide, whereas Bcl-2 targeted to the outer mitochondrial membrane protects against all these stimuli (Annis *et al*, 2001; Rudner *et al*, 2001). Conversely, transient overexpression of Bcl-2 targeted to mitochondria has been shown to have pro-apoptotic activity (Wang *et al*, 2001). In view of this, it would be of interest in the future to study the subcellular localisation of Bcl-2 in our stably transfected T3M-4 cells. However, based on the results shown here, we can conclude that the majority of Bcl-2 localises in a favourable way, as it effectively inhibits apoptosis induced by 9cRA.

Interestingly, the broad-range caspase inhibitors Z-VAD.fmk and Boc-D.fmk were shown to inhibit nuclear fragmentation associated with apoptosis induced by 9cRA and still had no effect on over-all cell viability, identified by mitochondrial activity. This suggests that caspase activation is involved in retinoid-induced apoptosis in pancreatic adenocarcinoma cells, but that other mechanisms can substitute for this activation and execute cell death. The subject of caspase-independent apoptosis has been reviewed by Borner and Monney (1999), and they point out that although inhibitors like Z-VAD.fmk effectively block cleavage of caspase substrates as well as nuclear fragmentation, morphological and mitochondrial changes still occur in most systems and the cells eventually die, possibly through activation of alternative proteases. The possibility that the effect of 9cRA would be delayed, although not inhibited, by addition of the caspase inhibitors was considered, but discarded as no effect on mitochondrial activity was seen at any time-point studied. It has been reported that retinoic acids can have direct effects on mitochondria, causing a fall in transmembrane potential, organelle swelling and cytochrome *c* release (Rigobello *et al*, 1999). These are interesting issues, which merit further investigation but are beyond the scope of this paper.

A major aim of this study was to investigate the role of different retinoid receptor subtypes in activation of the apoptotic response to the pan-agonist 9cRA. We determined that the four cell lines studied showed similar receptor expression patterns, with one important exception. That is, 9cRA-resistant A818-4 cells express almost undetectable levels of RAR-γ. RAR-α, RAR-γ and RXR-α were expressed in all cells but weak expression of RAR-β could be detected in T3M-4 cells only. This is in agreement with previous reports, which have shown that RAR-β expression is generally low or absent in pancreatic cancer cells (Kaiser *et al*, 1997). Although induction of RAR-β has been reported to be an indicator of retinoid response (Seewaldt *et al*, 1995; Lee *et al*, 2000; Sun *et al*, 2000), we could detect no induction of either RAR subtype upon retinoid treatment. This may seem surprising, but is likely to be due to methylation of the RAR-beta gene (Ueki *et al*, 2000).

Among the receptor subtype selective compounds tested, only two had inhibitory activity as single agents. LG101093, which binds selectively to RAR-γ and the pan RAR-agonist LGD1550 both caused significantly reduced cell viability and induced nuclear fragmentation, characteristic of apoptosis. Furthermore, an RAR-β/γ selective antagonist (LG030403) was shown to counteract the effect of LG101093, LGD1550, as well as 9cRA. Taken together, these results demonstrate that activation of RAR-γ is essential for induction of apoptosis by retinoids in pancreatic adenocarcinoma cells. This is also supported by the fact that A818-4 are resistant to the effects of 9cRA. A number of earlier studies have established a connection between RAR-γ and retinoid induced growth arrest and apoptosis in various cell types. Expression of RAR-β is often lost or decreased in tumour cells (Seewaldt *et al*, 1995; Wan *et al*, 1999; Lee *et al*, 2000; Sun *et al*, 2000). However, results from a study in melanoma cells showed that, although RAR-β expression was induced by activation of any of the RAR or RXR subtypes, only RAR-γ selective compounds were able to induce differentiation followed by apoptosis. This suggests a critical role for RAR-γ in apoptosis (Spanjaard *et al*, 1997). The same finding has been observed in neuroblastoma cells. In contrast to ATRA, which induces differentiation in these cells, RAR-γ selective retinoids were shown to induce apoptotic cell death (Meister *et al*, 1998).

Pancreatic carcinomas, like many other tumours, show frequent loss of RAR-β expression and an association between this loss and development of pancreatic malignancy is supported by transfection experiments restoring RAR-β expression in pancreatic cancer cells (Kaiser *et al*, 1997). On the other hand, selective loss of RAR-γ expression in retinoid resistant cells (see Figures 1 and 5 and Rosewicz *et al*, 1995) implies an important role for this subtype in conferring sensitivity to retinoids. Kaiser *et al* (1998) demonstrated that reintroduction of RAR-γ1 into resistant cells could restore their ability to be growth inhibited in response to retinoic acid treatment. Results presented in this study also make it clear that activation of RAR-γ is necessary and sufficient for induction of apoptosis in human pancreatic cancer cells. Since the RXR agonist LG101305 alone had no effect on cell viability, RXR homodimers do not mediate the response. Instead, the fact that the effects of LG101093 and LGD1550 are potentiated by LG101305 supports a model where RAR/RXR heterodimers are optimally activated when both RAR and RXR bind their ligand (Apfel *et al*, 1995; Minucci *et al*, 1997). However, RXR activation is clearly not required, since this potentiation by LG101305 was abolished at higher concentrations of LG101093 and LGD1550.

In summary, retinoids activating RARs, and RAR-γ in particular, induce apoptosis in pancreatic adenocarcinoma cells via a pathway involving altered expression of Bcl-2 family members as well as caspase activation. Together with our previous report that 9cRA enhances the effect of currently used chemotherapy (Pettersson *et al*, 2001), and promising clinical data from a phase II trial of 13-*cis* retinoic acid and interferon-α (Brembeck *et al*, 1998), this encourages further work evaluating the use of receptor subtype-selective retinoids in management of this disease.
